# 5-Ethyl-2-methyl-3-phenyl­sulfonyl-1-benzofuran

**DOI:** 10.1107/S1600536808012877

**Published:** 2008-05-07

**Authors:** Hong Dae Choi, Pil Ja Seo, Byeng Wha Son, Uk Lee

**Affiliations:** a Department of Chemistry, Dongeui University, San 24 Kaya-dong, Busanjin-gu, Busan 614-714, Republic of Korea; b Department of Chemistry, Pukyong National University, 599-1 Daeyeon 3-dong, Nam-gu, Busan 608-737, Republic of Korea

## Abstract

The title compound, C_17_H_16_O_3_S, was prepared by the oxidation of 5-ethyl-2-methyl-3-phenyl­sulfanyl-1-benzofuran with 3-chloro­peroxy­benzoic acid. The phenyl ring makes a dihedral angle of 75.94 (8)° with the plane of the benzofuran fragment. The crystal structure is stabilized by π–π inter­actions between the furan rings of neighbouring mol­ecules [centroid–centroid distance = 3.620 (4) Å]. In addition, the crystal structure exhibits C—H⋯π inter­actions.

## Related literature

For the crystal structures of similar 3-phenyl­sulfonyl-1-benzofuran compounds, see: Choi *et al.* (2008*a* ▶,*b* ▶).
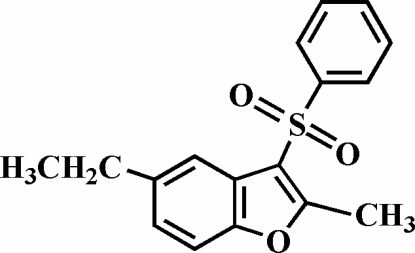


## Experimental

### 

#### Crystal data

C_17_H_16_O_3_S
*M* *_r_* = 300.36
Monoclinic, 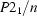

*a* = 8.7009 (4) Å
*b* = 8.2019 (4) Å
*c* = 20.682 (1) Å
β = 97.301 (1)°
*V* = 1463.98 (12) Å^3^
*Z* = 4
Mo *K*α radiation
μ = 0.23 mm^−1^
*T* = 173 (2) K
0.40 × 0.20 × 0.20 mm

#### Data collection

Bruker SMART CCD diffractometer
Absorption correction: none
8693 measured reflections
3194 independent reflections
2264 reflections with *I* > 2σ(*I*)
*R*_int_ = 0.048

#### Refinement

*R*[*F* ^2^ > 2σ(*F* ^2^)] = 0.056
*wR*(*F* ^2^) = 0.148
*S* = 1.03
3194 reflections
192 parameters
H-atom parameters constrained
Δρ_max_ = 0.42 e Å^−3^
Δρ_min_ = −0.49 e Å^−3^

### 

Data collection: *SMART* (Bruker, 2001 ▶); cell refinement: *SAINT* (Bruker, 2001 ▶); data reduction: *SAINT*; program(s) used to solve structure: *SHELXS97* (Sheldrick, 2008 ▶); program(s) used to refine structure: *SHELXL97* (Sheldrick, 2008 ▶); molecular graphics: *ORTEP-3* (Farrugia, 1997 ▶) and *DIAMOND* (Brandenburg, 1998 ▶); software used to prepare material for publication: *SHELXL97*.

## Supplementary Material

Crystal structure: contains datablocks global, I. DOI: 10.1107/S1600536808012877/cf2194sup1.cif

Structure factors: contains datablocks I. DOI: 10.1107/S1600536808012877/cf2194Isup2.hkl

Additional supplementary materials:  crystallographic information; 3D view; checkCIF report

## Figures and Tables

**Table 1:** Hydrogen-bond geometry (Å, °)

*D*—H⋯*A*	*D*—H	H⋯*A*	*D*⋯*A*	*D*—H⋯*A*
C15—H15*C*⋯*Cg*2^i^	0.98	2.80	3.592 (4)	139
C16—H16*B*⋯*Cg*2^ii^	0.98	3.21	3.903 (4)	128

Symmetry codes: (i) 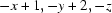; (ii) 
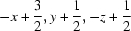
.

**Fig. 1.**
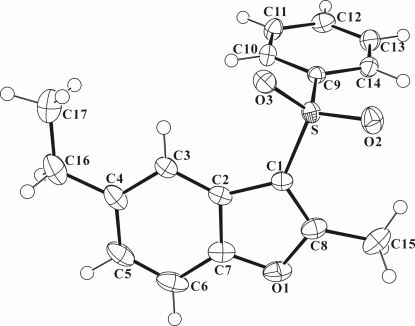
The molecular structure of the title compound, showing displacement ellipsoids drawn at the 50% probability level.

**Fig. 2.**
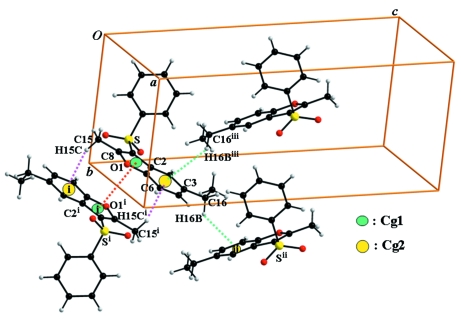
π—π and C—H···π interactions (dotted lines) in the title compound. Cg denotes the ring centroids. [Symmetry code: (i) -x + 1, -y + 2, -z: (ii) -x + 3/2, y + 1/2, -z + 1/2; (iii) -x + 3/2, y - 1/2, -z + 1/2.]

## supplementary crystallographic information

### Comment

As a part of our ongoing studies on the synthesis and structure of 3-phenyl-sulfonyl-1-benzofuran analogues, the crystal structure of 5-bromo-2-methyl-3-phenylsulfonyl-1-benzofuran (Choi *et al.*, 2008*a*) and 2,5,7-trimethyl-3-phenylsulfonyl-1-benzofuran (Choi *et al.*, 2008*b*) have been described in the literature. Here we report the crystal structure of the title compound, 5-ethyl-2-methyl-3-phenylsulfonyl-1-benzofuran (Fig. 1).

The benzofuran unit is essentially planar, with a mean deviation of 0.007 Å from the least-squares plane defined by the nine constituent atoms. The phenyl ring (C9—C14) makes a dihedral angle of 75.94 (8)° with the plane of the benzofuran fragment. The crystal packing (Fig. 2) is stabilized by aromatic π-π stacking interactions between the furan rings of neighbouring molecules. The *Cg*1···*Cg*1^i^ distance is 3.620 (4) Å (*Cg*1 is the centroid of the O1/C8/C1/C2/C7 furan ring, symmetry code as in Fig. 2). The molecular packing is further stabilized by C—H···π interactions (Table 1 and Fig. 2); one between a methyl H atom and the benzene ring of the benzofuran unit, *i.e.* C15—H15C···*Cg*2^i^, a second between a methylene H atom of the ethyl group and the benzene ring of the benzofuran unit, *i.e.* C16—H16B···*Cg*2^ii^, respectively. In both cases the benzene ring (*Cg*2) is involved (*Cg*2 is the centroid of the C2–7 benzene ring, symmetry code as in Fig. 2).

### Experimental

3-Chloroperoxybenzoic acid (77%, 471 mg, 2.1 mmol) was added in small portions to a stirred solution of 5-ethyl-2-methyl-3-phenylsulfanyl-1-benzofuran (268 mg, 1.0 mmol) in dichloromethane (30 ml) at 273 K. After being stirred for 4 h at room temperature, the mixture was washed with saturated sodium bicarbonate solution and the organic layer was separated, dried over magnesium sulfate, filtered and concentrated in vacuum. The residue was purified by column chromatography (hexane-ethyl acetate, 2:1 *v*/*v*) to afford the title compound as a colorless solid [yield 80%, m.p. 396- 397 K; *R*_f_ = 0.66 (hexane-ethyl acetate, 2:1 *v*/*v*)]. Single crystals suitable for X-ray diffraction were prepared by evaporation of a solution of the title compound in benzene at room temperature. Spectroscopic analysis: ^1^H NMR (CDCl_3_, 400 MHz) δ 1.26 (t, J = 7.68 Hz, 3H), 2.74 (q, J = 7.72 Hz, 2H), 2.79 (s, 3H), 7.14 (d, J = 8.44 Hz and 1.48 Hz, 1H), 7.32 (d, J = 8.44 Hz, 1H), 7.48–7.61 (m, 3H), 7.69 (s, 1H), 8.01 (d, J = 6.96 Hz, 2H); EI—MS 300 [*M*^+^].

### Refinement

All H atoms were positioned geometrically and refined using a riding model, with C—H = 0.95 Å for aromatic H atoms, 0.99 Å for methylene H atoms and 0.98 Å for methyl H atoms, respectively, and with *U*_iso_(H) = 1.2U_eq_(C) for aromatic and methylene H atoms, and 1.5U_eq_(C) for methyl H atoms.

### Crystal data

**Table:**

C_17_H_16_O_3_S	*F*_000_ = 632
*M**_r_* = 300.36	*D*_x_ = 1.363 Mg m^−^^3^
Monoclinic, *P*2_1_/*n*	Mo *K*α radiation λ = 0.71073 Å
Hall symbol: -P_2yn	Cell parameters from 2779 reflections
*a* = 8.7009 (4) Å	θ = 2.4–27.9º
*b* = 8.2019 (4) Å	µ = 0.23 mm^−^^1^
*c* = 20.682 (1) Å	*T* = 173 (2) K
β = 97.301 (1)º	Block, colorless
*V* = 1463.98 (12) Å^3^	0.40 × 0.20 × 0.20 mm
*Z* = 4	

### Data collection

**Table:**

Bruker SMART CCD diffractometer	3194 independent reflections
Radiation source: fine-focus sealed tube	2264 reflections with *I* > 2σ(*I*)
Monochromator: graphite	*R*_int_ = 0.048
Detector resolution: 10.0 pixels mm^-1^	θ_max_ = 27.0º
*T* = 173(2) K	θ_min_ = 2.4º
φ and ω scans	*h* = −8→11
Absorption correction: none	*k* = −8→10
8693 measured reflections	*l* = −24→26

### Refinement

**Table:**

Refinement on *F*^2^	Secondary atom site location: difference Fourier map
Least-squares matrix: full	Hydrogen site location: inferred from neighbouring sites
*R*[*F*^2^ > 2σ(*F*^2^)] = 0.056	H-atom parameters constrained
*wR*(*F*^2^) = 0.148	*w* = 1/[σ^2^(*F*_o_^2^) + (0.0725*P*)^2^ + 0.989*P*] where *P* = (*F*_o_^2^ + 2*F*_c_^2^)/3
*S* = 1.03	(Δ/σ)_max_ < 0.001
3194 reflections	Δρ_max_ = 0.42 e Å^−^^3^
192 parameters	Δρ_min_ = −0.49 e Å^−^^3^
Primary atom site location: structure-invariant direct methods	Extinction correction: none

### Special details

**Table:**

Geometry. All e.s.d.'s (except the e.s.d. in the dihedral angle between two l.s. planes) are estimated using the full covariance matrix. The cell e.s.d.'s are taken into account individually in the estimation of e.s.d.'s in distances, angles and torsion angles; correlations between e.s.d.'s in cell parameters are only used when they are defined by crystal symmetry. An approximate (isotropic) treatment of cell e.s.d.'s is used for estimating e.s.d.'s involving l.s. planes.
Refinement. Refinement of *F*^2^ against ALL reflections. The weighted *R*-factor *wR* and goodness of fit *S* are based on *F*^2^, conventional *R*-factors *R* are based on *F*, with *F* set to zero for negative *F*^2^. The threshold expression of *F*^2^ > σ(*F*^2^) is used only for calculating *R*-factors(gt) *etc*. and is not relevant to the choice of reflections for refinement. *R*-factors based on *F*^2^ are statistically about twice as large as those based on *F*, and *R*- factors based on ALL data will be even larger.

### Fractional atomic coordinates and isotropic or equivalent isotropic displacement parameters (Å^2^)

**Table:**

	*x*	*y*	*z*	*U*_iso_*/*U*_eq_	
S	0.22072 (7)	0.75221 (8)	0.07999 (3)	0.01957 (19)	
O1	0.6098 (2)	0.7989 (3)	0.00433 (10)	0.0340 (5)	
O2	0.1125 (2)	0.7474 (2)	0.02135 (9)	0.0302 (5)	
O3	0.1953 (2)	0.8653 (2)	0.13092 (9)	0.0256 (4)	
C1	0.4056 (3)	0.7908 (3)	0.06010 (12)	0.0206 (6)	
C2	0.5331 (3)	0.8631 (3)	0.10261 (13)	0.0219 (6)	
C3	0.5561 (3)	0.9250 (3)	0.16565 (13)	0.0253 (6)	
H3	0.4738	0.9264	0.1918	0.030*	
C4	0.7023 (3)	0.9852 (4)	0.18992 (15)	0.0318 (7)	
C5	0.8208 (3)	0.9848 (4)	0.15001 (17)	0.0362 (8)	
H5	0.9195	1.0266	0.1671	0.043*	
C6	0.8006 (3)	0.9267 (4)	0.08721 (16)	0.0361 (8)	
H6	0.8817	0.9286	0.0605	0.043*	
C7	0.6548 (3)	0.8648 (4)	0.06503 (14)	0.0286 (6)	
C8	0.4583 (3)	0.7531 (3)	0.00219 (13)	0.0283 (6)	
C9	0.2322 (3)	0.5547 (3)	0.11444 (12)	0.0184 (5)	
C10	0.3091 (3)	0.5341 (3)	0.17728 (13)	0.0246 (6)	
H10	0.3552	0.6245	0.2010	0.030*	
C11	0.3172 (3)	0.3800 (4)	0.20437 (13)	0.0280 (6)	
H11	0.3697	0.3637	0.2470	0.034*	
C12	0.2487 (3)	0.2487 (3)	0.16934 (14)	0.0294 (6)	
H12	0.2542	0.1429	0.1882	0.035*	
C13	0.1722 (3)	0.2713 (3)	0.10688 (14)	0.0280 (6)	
H13	0.1255	0.1809	0.0833	0.034*	
C14	0.1637 (3)	0.4242 (3)	0.07899 (13)	0.0236 (6)	
H14	0.1119	0.4400	0.0362	0.028*	
C15	0.3901 (4)	0.6744 (4)	−0.05890 (14)	0.0389 (8)	
H15A	0.2823	0.6446	−0.0556	0.058*	
H15B	0.4494	0.5762	−0.0664	0.058*	
H15C	0.3933	0.7503	−0.0953	0.058*	
C16	0.7384 (4)	1.0484 (5)	0.25879 (18)	0.0546 (10)	
H16A	0.8332	0.9924	0.2791	0.066*	
H16B	0.7639	1.1657	0.2561	0.066*	
C17	0.6211 (5)	1.0323 (6)	0.30328 (18)	0.0597 (11)	
H17A	0.5272	1.0912	0.2855	0.090*	
H17B	0.6612	1.0782	0.3459	0.090*	
H17C	0.5965	0.9168	0.3083	0.090*	

### Atomic displacement parameters (Å^2^)

**Table:**

	*U*^11^	*U*^22^	*U*^33^	*U*^12^	*U*^13^	*U*^23^
S	0.0181 (3)	0.0187 (3)	0.0212 (3)	0.0015 (2)	−0.0004 (2)	0.0000 (3)
O1	0.0302 (11)	0.0407 (13)	0.0338 (12)	0.0061 (9)	0.0137 (9)	0.0073 (10)
O2	0.0274 (10)	0.0313 (11)	0.0295 (10)	0.0013 (8)	−0.0058 (8)	0.0035 (9)
O3	0.0231 (10)	0.0222 (10)	0.0323 (11)	0.0004 (8)	0.0063 (8)	−0.0056 (8)
C1	0.0222 (13)	0.0177 (13)	0.0225 (13)	0.0017 (10)	0.0049 (10)	0.0029 (11)
C2	0.0177 (13)	0.0195 (13)	0.0289 (14)	0.0024 (10)	0.0045 (11)	0.0070 (11)
C3	0.0202 (14)	0.0259 (15)	0.0299 (15)	−0.0016 (11)	0.0037 (11)	0.0014 (12)
C4	0.0241 (15)	0.0291 (16)	0.0404 (17)	−0.0061 (12)	−0.0025 (12)	0.0039 (13)
C5	0.0175 (14)	0.0372 (18)	0.052 (2)	−0.0044 (12)	−0.0009 (13)	0.0117 (16)
C6	0.0208 (15)	0.0384 (18)	0.051 (2)	0.0047 (12)	0.0116 (14)	0.0179 (16)
C7	0.0264 (15)	0.0277 (15)	0.0329 (15)	0.0067 (12)	0.0082 (12)	0.0084 (13)
C8	0.0306 (15)	0.0293 (15)	0.0256 (14)	0.0069 (12)	0.0058 (12)	0.0089 (13)
C9	0.0171 (12)	0.0192 (13)	0.0191 (13)	−0.0002 (10)	0.0037 (10)	−0.0006 (10)
C10	0.0263 (14)	0.0255 (15)	0.0212 (13)	−0.0015 (11)	−0.0001 (11)	−0.0037 (11)
C11	0.0324 (16)	0.0306 (16)	0.0198 (13)	0.0005 (12)	−0.0008 (11)	0.0012 (12)
C12	0.0373 (16)	0.0208 (14)	0.0317 (15)	0.0006 (12)	0.0104 (13)	0.0044 (13)
C13	0.0333 (15)	0.0224 (15)	0.0289 (15)	−0.0044 (11)	0.0062 (12)	−0.0055 (12)
C14	0.0258 (14)	0.0239 (14)	0.0209 (13)	−0.0016 (11)	0.0023 (11)	−0.0019 (11)
C15	0.052 (2)	0.0423 (19)	0.0232 (15)	0.0102 (16)	0.0090 (14)	−0.0009 (14)
C16	0.0393 (19)	0.066 (3)	0.058 (2)	−0.0246 (18)	0.0013 (17)	−0.017 (2)
C17	0.059 (2)	0.077 (3)	0.042 (2)	−0.018 (2)	−0.0003 (18)	−0.019 (2)

### Geometric parameters (Å, °)

**Table:**

S—O2	1.4386 (19)	C9—C10	1.395 (3)
S—O3	1.4417 (19)	C10—C11	1.381 (4)
S—C1	1.740 (3)	C10—H10	0.950
S—C9	1.767 (3)	C11—C12	1.389 (4)
O1—C8	1.367 (3)	C11—H11	0.950
O1—C7	1.377 (4)	C12—C13	1.388 (4)
C1—C8	1.371 (4)	C12—H12	0.950
C1—C2	1.451 (4)	C13—C14	1.379 (4)
C2—C3	1.390 (4)	C13—H13	0.950
C2—C7	1.391 (4)	C14—H14	0.950
C3—C4	1.397 (4)	C15—H15A	0.980
C3—H3	0.950	C15—H15B	0.980
C4—C5	1.400 (4)	C15—H15C	0.980
C4—C16	1.511 (5)	C16—C17	1.464 (5)
C5—C6	1.374 (5)	C16—H16A	0.990
C5—H5	0.950	C16—H16B	0.990
C6—C7	1.389 (4)	C17—H17A	0.980
C6—H6	0.950	C17—H17B	0.980
C8—C15	1.474 (4)	C17—H17C	0.980
C9—C14	1.388 (4)		
			
O2—S—O3	119.24 (12)	C11—C10—C9	118.9 (2)
O2—S—C1	109.38 (12)	C11—C10—H10	120.6
O3—S—C1	106.76 (12)	C9—C10—H10	120.6
O2—S—C9	108.19 (12)	C10—C11—C12	120.1 (3)
O3—S—C9	107.54 (11)	C10—C11—H11	119.9
C1—S—C9	104.81 (12)	C12—C11—H11	119.9
C8—O1—C7	107.4 (2)	C11—C12—C13	120.3 (3)
C8—C1—C2	107.9 (2)	C11—C12—H12	119.8
C8—C1—S	126.0 (2)	C13—C12—H12	119.8
C2—C1—S	126.13 (19)	C14—C13—C12	120.3 (3)
C3—C2—C7	119.4 (2)	C14—C13—H13	119.9
C3—C2—C1	136.5 (2)	C12—C13—H13	119.9
C7—C2—C1	104.1 (2)	C13—C14—C9	118.9 (2)
C2—C3—C4	118.8 (3)	C13—C14—H14	120.5
C2—C3—H3	120.6	C9—C14—H14	120.5
C4—C3—H3	120.6	C8—C15—H15A	109.5
C3—C4—C5	119.5 (3)	C8—C15—H15B	109.5
C3—C4—C16	122.0 (3)	H15A—C15—H15B	109.5
C5—C4—C16	118.4 (3)	C8—C15—H15C	109.5
C6—C5—C4	122.9 (3)	H15A—C15—H15C	109.5
C6—C5—H5	118.6	H15B—C15—H15C	109.5
C4—C5—H5	118.6	C17—C16—C4	118.9 (3)
C5—C6—C7	116.2 (3)	C17—C16—H16A	107.6
C5—C6—H6	121.9	C4—C16—H16A	107.6
C7—C6—H6	121.9	C17—C16—H16B	107.6
O1—C7—C6	125.9 (3)	C4—C16—H16B	107.6
O1—C7—C2	110.9 (2)	H16A—C16—H16B	107.0
C6—C7—C2	123.2 (3)	C16—C17—H17A	109.5
O1—C8—C1	109.7 (2)	C16—C17—H17B	109.5
O1—C8—C15	115.4 (2)	H17A—C17—H17B	109.5
C1—C8—C15	134.8 (3)	C16—C17—H17C	109.5
C14—C9—C10	121.5 (2)	H17A—C17—H17C	109.5
C14—C9—S	119.7 (2)	H17B—C17—H17C	109.5
C10—C9—S	118.85 (19)		
			
O2—S—C1—C8	−26.7 (3)	C1—C2—C7—C6	179.3 (3)
O3—S—C1—C8	−157.0 (2)	C7—O1—C8—C1	0.8 (3)
C9—S—C1—C8	89.1 (3)	C7—O1—C8—C15	−178.2 (2)
O2—S—C1—C2	155.7 (2)	C2—C1—C8—O1	−1.1 (3)
O3—S—C1—C2	25.4 (3)	S—C1—C8—O1	−179.00 (19)
C9—S—C1—C2	−88.5 (2)	C2—C1—C8—C15	177.7 (3)
C8—C1—C2—C3	−179.7 (3)	S—C1—C8—C15	−0.2 (5)
S—C1—C2—C3	−1.8 (5)	O2—S—C9—C14	10.3 (2)
C8—C1—C2—C7	0.9 (3)	O3—S—C9—C14	140.3 (2)
S—C1—C2—C7	178.8 (2)	C1—S—C9—C14	−106.4 (2)
C7—C2—C3—C4	−1.0 (4)	O2—S—C9—C10	−169.12 (19)
C1—C2—C3—C4	179.6 (3)	O3—S—C9—C10	−39.1 (2)
C2—C3—C4—C5	1.3 (4)	C1—S—C9—C10	74.3 (2)
C2—C3—C4—C16	−177.6 (3)	C14—C9—C10—C11	0.2 (4)
C3—C4—C5—C6	−0.3 (5)	S—C9—C10—C11	179.6 (2)
C16—C4—C5—C6	178.6 (3)	C9—C10—C11—C12	−0.4 (4)
C4—C5—C6—C7	−0.9 (5)	C10—C11—C12—C13	0.2 (4)
C8—O1—C7—C6	−179.9 (3)	C11—C12—C13—C14	0.1 (4)
C8—O1—C7—C2	−0.2 (3)	C12—C13—C14—C9	−0.3 (4)
C5—C6—C7—O1	−179.2 (3)	C10—C9—C14—C13	0.1 (4)
C5—C6—C7—C2	1.2 (4)	S—C9—C14—C13	−179.2 (2)
C3—C2—C7—O1	−179.9 (2)	C3—C4—C16—C17	5.8 (6)
C1—C2—C7—O1	−0.4 (3)	C5—C4—C16—C17	−173.1 (3)
C3—C2—C7—C6	−0.3 (4)		

### Hydrogen-bond geometry (Å, °)

**Table:**

*D*—H···*A*	*D*—H	H···*A*	*D*···*A*	*D*—H···*A*
C15—H15C···Cg2^i^	0.98	2.80	3.592 (4)	139
C16—H16B···Cg2^ii^	0.98	3.21	3.903 (4)	128

Symmetry codes: (i) −*x*+1, −*y*+2, −*z*; (ii) −*x*+3/2, *y*+1/2, −*z*+1/2.
